# Screening for Obstructive Sleep Apnea Among the Adult Population in the Northeastern Region of Romania

**DOI:** 10.3390/dj13050208

**Published:** 2025-05-12

**Authors:** Olja Tanellari, Adela Alushi, Celiana Toti, Carina Balcos, Adina Oana Armencia, Tinela Panaite, Irina Zetu

**Affiliations:** 1 Department of Orthodontics, Faculty of Dental Medicine, “Grigore T. Popa” University of Medicine and Pharmacy, 700115 Iasi, Romania; oli_koca@yahoo.com (O.T.); tinela-panaite@umfiasi.ro (T.P.); nicoletazetu@gmail.com (I.Z.); 2 Department of Orthodontics, Aldent University, 1005 Tirana, Albania; adela.alushi@ual.edu.al; 3 Department of Orthodontics, Faculty of Dental Medicine, University of Medicine, Tirana (UMT), 1005 Tirana, Albania; celjana.toti@umed.edu.al; 4 Department of Surgery, Faculty of Dental Medicine, “Grigore T. Popa” University of Medicine and Pharmacy, 700115 Iasi, Romania; carina.balcos@umfiasi.ro

**Keywords:** obstructive sleep apnea, screening, STOP-Bang questionnaire, Epworth questionnaire

## Abstract

**Background**: Sleep apnea is a breathing affliction that affects sleep quality, with significant implications for overall physical and oral health, as well as mental health. **Aim**: The aim of this study was to evaluate the validity of the STOP-Bang and Epworth questionnaires as preoperative screening tools for obstructive sleep apnea (OSA) in the adult population from the NE region of Romania. **Materials and Methods**: A total of 222 participants were selected. The questionnaire method was used, with the subjects completing the STOP-Bang and Epworth questionnaires. A chi-squared test, an ANOVA, and Student’s *t*-tests were used for the statistical analysis. **Results**: the STOP-Bang questionnaire revealed an increased risk of OSA among those with a high BMI, an age over 50 years, or a large neck circumference. Regarding the Epworth questionnaire, daytime sleepiness was more frequent among obese individuals and those with associated pathologies. Significant correlations were found between OSA and obesity, age, and associated pathologies, with limited observations on the influence of gender on the risk of OSA. **Conclusions**: The studied questionnaires are effective and easy-to-use tools for the preoperative screening of OSA, demonstrating a significant correlation between the identified risk factors and the disease severity.

## 1. Introduction

Obstructive sleep apnea (OSA) is marked by repeated airflow cessation during sleep due to upper airway obstruction, leading to frequent arousals as the patient struggles to breathe. Early screening is essential, as undiagnosed OSA can be worsened by analgesic, anesthetic, and sedative drugs. The risk factors include age, the male gender, menopause, anatomical anomalies, heredity, smoking, alcohol use, and obesity. If left untreated, OSA can cause symptoms such as nocturnal choking, morning headaches, and daytime sleepiness, and is associated with cardiovascular disease, depression, and other health conditions [1,2,3,4].

OSA is associated with serious outcomes such as cardiovascular disease, diabetes, and an increased mortality [1,5,6,7,8,9,10,11,12,13,14,15,16,17]. Despite a prevalence of 2–7%, it remains widely underdiagnosed—especially in surgical patients—raising postoperative risks [3,4,5,6,7,8,9,10,11,12].

The early screening for OSA, including in dental practices, is essential and can be effectively carried out using simple tools such as the STOP-Bang questionnaire, STOP, Berlin, and the P-SAP score [11,12,18,19]. Given the increased risk of perioperative complications in undiagnosed patients [7,20,21,22], the use of the STOP-Bang questionnaire is particularly valuable, as it combines symptom-based and demographic elements, offering a faster and more practical screening approach compared to other tools [23,24,25]. So, the aim of this study was to determine the validity of the STOP-Bang and Epworth questionnaires as preoperative screening tools by identifying individuals who are predisposed to developing a severe form of OSA in the population from the NE region of Romania.

## 2. Materials and Methods

### 2.1. Study Design and Population

A cross-sectional study was conducted between January 2022 and January 2023, with the approval of the Institutional Ethics Committee of the “Grigore T. Popa” University of Medicine and Pharmacy in Iași, Romania (No. 188/25 May 2022), in accordance with the Hel Declaration. The initial group consisted of 350 patients attending private dental practices in the northeastern region of Romania. The following inclusion criteria were used to select the study participants: patients willing to complete the questionnaires, those over 40 years of age, both females and males, and those presenting risk factors for obstructive sleep apnea (increased weight, persistent drowsiness and fatigue, unsatisfactory sleep, etc.). Incomplete questionnaires, patients who did not present risk factors for sleep apnea, and participants who did not live in the northeastern region of Romania were excluded from this study.

At the beginning of the survey, the participants were provided with information about the research and its objectives. The completion of the questionnaire was considered an indication of consent to participate. Participation in the study was entirely voluntary and anonymous, and no identifying data were collected.

From the patient’s chart, we collected data related to the patient’s type/class of dento-maxillary anomaly, their neck circumference measured in cm, and their body mass index (BMI). BMI is a measure of body fat according to height and weight, applicable to both men and women. The BMI classifications are as follows: underweight (<18.5), normal weight (18.5–24.9), overweight (25–29.9), and obese (≥30).

### 2.2. Instruments

Two questionnaires were used to collect data:

#### 2.2.1. The STOP-Bang Questionnaire

The STOP-Bang questionnaire is a sensitive and easy-to-administer screening tool for OSA [6]. Originally applied for preoperative screening, the STOP-Bang questionnaire has been subsequently validated in various populations [26,27,28,29].

The questionnaire contains 8 questions that are answered with “Yes”/”No”. The risk of OSA is considered low when 0–2 questions are answered positively. The risk is considered medium when the subject answers positively to 3–4 questions and high when the participant selects the answer of “Yes” for 5–8 questions; also, the risk is increased when the subject answers “Yes” to 2 or more of the first 4 questions; in male subjects who answer “Yes” to 2 or more of the first 4 questions when associated with a BMI > 35 kg/m^2^; or “Yes” to 2 or more of the first 4 questions associated with neck circumference (43 cm in men, 41 cm in women) [26,27,28,29].

#### 2.2.2. Epworth Questionnaire—Daytime Sleepiness Scale (ESS)

The Epworth sleepiness scale (ESS) assesses excessive daytime sleepiness—a symptom due to its direct impact on quality of life and the risk of road accidents, and an indicator of poor sleep quality in patients with OSA [29,30,31,32].

The questionnaire contains 8 questions that participants must answer by choosing an answer option as follows: 0 = do not doze/fall asleep, 1 = low probability of dozing/falling asleep, 2 = moderate probability of dozing/falling asleep, or 3 = high probability of dozing/falling asleep. The final score represents the sum of the chosen scores. A score between 10 and 17 points indicates pathological drowsiness, and a score higher than 18 is recorded in subjects with a state of accentuated drowsiness. If the score obtained in this test exceeds the value of 10, consultation with a sleep specialist is recommended [32].

### 2.3. Statistical Analysis

For the data analysis, the SPSS 26.0 for Windows program was used, with the aim of highlighting the results, which are presented in the form of frequencies, mean values, and standard deviations. An ANOVA and Student’s *t*-test were used for the comparison of variables. The statistical significance was set at a value of *p* = 0.05.

## 3. Results

### 3.1. Participant Characteristics

Of the 350 questionnaires distributed, only 222 questionnaires were submitted correctly and completely. The analysis of demographic data indicated that 59% of these participants were male subjects and the mean age of the group was 61.25 years (SD = 7.165) (minimum age: 42 years, maximum age: 75 years). More than half of the subjects came from urban areas (68%) and 56.3% of them were of a high socio-economic level (Table 1).

### 3.2. Analysis of the STOP-Bang Questionnaire

The STOP-Bang responses indicated strong associations with obesity and comorbidities, while gender showed minimal influence. Loud snoring, daytime sleepiness, and sleep apnea were significantly more common in obese individuals and those with comorbidities (*p* < 0.01). Hypertension was linked to comorbidities (*p* = 0.009), and an age > 50 was correlated with a high BMI and comorbidities (*p* = 0.000). A neck circumference > 41 cm was more frequent in men, obese subjects, and those with comorbidities. A high OSA risk was strongly associated with obesity (74.7%) and comorbidities (56%) (Table 2).

Multinomial regression (Table 3) identified the female gender (β = 0.80, *p* = 0.000), an age under 50 (β = 5.45, *p* = 0.000), and BMI (β = 10.1, *p* = 0.000) as significant predictors of OSA. Age and BMI significantly influenced the OSA risk. Individuals over 50 had a 5.5-fold higher likelihood of OSA (Exp(B) = 5.455, CI: 1.614–18.441, *p* = 0.006), while age had no significant impact on the medium-risk STOP-Bang group (*p* = 0.062). Overweight individuals (BMI of 25–30) had a 1.7-fold increased OSA risk, rising to over 10-fold in obese subjects (BMI ≥ 30; Exp(B) = 10.147, CI: 4.422–23.283, *p* = 0.000).

### 3.3. Epworth Questionnaire (ESS) Analysis

An analysis of the responses regarding the likelihood of dozing off in everyday situations revealed significant associations with BMI and comorbidities, while gender showed a less consistent influence. When reading, 62.6% of women and 38.2% of men reported a moderate chance of dozing off, which was significantly correlated with obesity (39.7%) and comorbid conditions (27.1%, *p* = 0.000). Watching TV was associated with moderate drowsiness in 56% of overweight and 63.7% of obese participants, with significant differences by gender, BMI, and comorbidities (*p* = 0.000).

In passive public environments, drowsiness was more frequent among women (78%), again linked to an increased BMI and comorbidities. During long car rides, 60.4% of women and 35.1% of men reported a high likelihood of dozing off, especially those with obesity and associated health issues (*p* = 0.000).

Post-meal drowsiness was also more common among women (62.6%), and was correlated with excess weight and comorbidities. Although less frequent, drowsiness while talking was significantly associated with BMI. Similarly, sleepiness during driving breaks was more commonly reported by individuals with an elevated BMI and comorbid conditions (*p* = 0.000) (Table 4).

**Figure 1:**
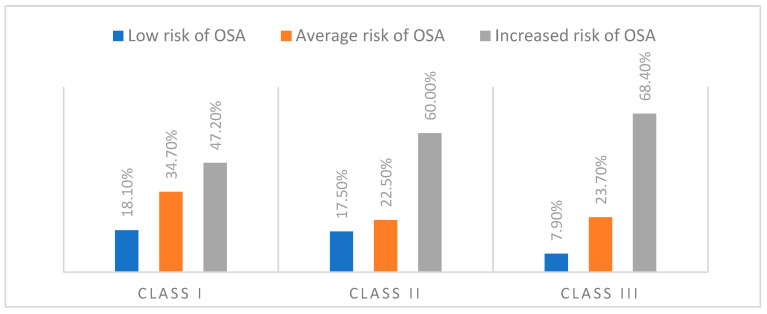
Distribution of subjects according to the risk calculated using the STOP-Bang questionnaire and the class of dento-maxillary anomaly.

The distribution of subjects by the obstructive sleep apnea (OSA) risk and the dento-maxillary anomaly (ADM) class, based on the STOP-Bang scores, showed a trend toward an increased OSA risk for classes II and III. Among those with class I ADM, 18.1% had a low risk, 34.7% had a medium risk, and 47.2% had a high risk. In class II, the proportion with a high risk rose to 60%, while low-risk cases decreased to 17.5%. In class III, a high risk was observed for 68.4% of the participants, with only 7.9% classified as low-risk (Figure 1).

**Figure 2:**
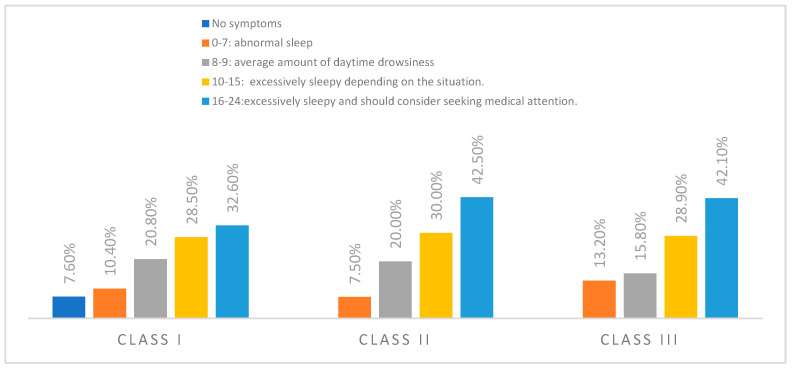
Distribution of subjects according to risk calculated using the Epworth questionnaire and dento-maxillary anomaly class.

An analysis of the distribution of daytime sleepiness across dento-maxillary anomaly (ADM) classes suggests a trend toward increased symptom severity with more advanced anomalies. The proportion of subjects reporting moderate sleepiness (scores 8–9) remained relatively stable across classes, ranging from 15.8% for class III to 20.8% for class I. Similarly, the percentage of those with low sleepiness (scores 0–7) was consistent across groups, with a slight increase observed for class III (13.2%) (Figure 2).

## 4. Discussion

While most OSA screening studies have focused on clinical populations, this study evaluated the predictive value of the STOP-Bang and Epworth tools in a general adult population from northeastern Romania—an underrepresented region in the literature. It also highlighted the underutilized role of dental professionals in OSA screening, offering a novel interdisciplinary perspective.

### 4.1. Key Predictive Risk Factors

This study confirmed the predictive relevance of key OSA risk factors, obesity, age, and an increased neck circumference—identified via the STOP-Bang questionnaire. Also, elevated Epworth scores further reflected excessive daytime sleepiness, a hallmark of OSA [25,31].

Several systemic conditions, including hypertension, diabetes, menopause, and skeletal anomalies, have been associated with an increased OSA risk [25,33,34,35,36,37,38,39]. The data from this study support the assertion that hypertension and other associated conditions (e.g., diabetes) are common among patients with OSA, particularly in its severe forms, and these associations are statistically significant.

Gender plays an important role in OSA. Although, in our study, gender showed a limited influence on the OSA risk according to the STOP-Bang scores, the regression analysis identified the female gender as a significant predictor, possibly due to differences in symptom reporting. Women often report symptoms such as insomnia, fatigue, and mood disturbances, largely influenced by hormonal changes [4,40,41,42,43]. In men over 55, OSA often coexists with obesity, diabetes, cardiovascular disease, and greater pharyngeal collapsibility [42,44,45,46].

Obesity is a major and bidirectional factor in OSA, and was confirmed by this study as the strongest predictor of the condition, influencing both the symptom severity and the overall risk, while also contributing to a cycle in which OSA promotes further weight gain through metabolic dysregulation and reduced physical activity, with a higher BMI and ENT-related disorders amplifying the disease severity [47,48,49,50,51].

### 4.2. Screening Tool Performance and Practicality

This study showed that both STOP-Bang and Epworth are effective screening tools for OSA, with STOP-Bang proving more efficient in identifying the overall risk and Epworth better reflecting the daytime symptom impact. STOP-Bang proved more efficient when the score differences were notable, especially since daytime symptoms like fatigue and reduced functioning often outweigh nocturnal signs in OSA patients [51].

Other studies have shown that the STOP-Bang questionnaire has strong predictive value, with scores above 3 indicating an increased perioperative risk and higher scores correlating with more severe OSA; despite varying item importance, its simplicity makes it an efficient and reliable screening tool [25,52,53,54,55].

### 4.3. Craniofacial Factors and OSA Risk

Cephalometric studies have shown that craniofacial anomalies such as micrognathia, mandibular retraction, and maxillary hypoplasia contribute to upper airway obstruction in OSA, particularly among non-obese individuals [56,57,58,59,60,61]. In this study, the OSA risk increased progressively from class I to class III of dento-maxillary anomalies, with a tendency toward greater daytime sleepiness in classes II and III, although without statistical significance. Class II malocclusions, which are associated with mandibular retraction, are especially linked to a reduced airway size [62,63], while class III anomalies may also compromise airway patency due to a skeletal imbalance [64].

A key strength of this study is its integration of OSA screening tools (STOP-Bang and Epworth) into dental practice. Dentists, who routinely evaluate craniofacial structures, are well positioned to detect the early anatomical signs of OSA—such as retrognathia or an increased neck circumference—thus supporting an interdisciplinary approach that may facilitate the early identification and referral of undiagnosed cases.

### 4.4. Study Limitations

This study had several limitations. The main constraint was the lack of objective diagnostic confirmation, as most participants were assessed using only self-reported tools (STOP-Bang and Epworth), without polysomnographic validation. Although these questionnaires are useful for identifying individuals at risk, they cannot replace a gold-standard sleep study. The study sample, composed only of adults over 40 from northeastern Romania, also limits the generalizability of the findings. Lastly, the cross-sectional design does not allow for causal conclusions, underlining the need for longitudinal research to assess risk progression over time.

### 4.5. Future Directions

Future research should aim to validate the STOP-Bang and Epworth screening tools by comparing their results with polysomnographic data in Romanian populations to determine their diagnostic accuracy. It is also important to expand the demographic and geographic scope of studies by including younger individuals and participants from other regions, thus improving the generalizability of the findings. Integrating OSA screening into routine dental practice should be explored, taking advantage of dentists’ accessibility for early detection. Additionally, longitudinal studies are needed to monitor the outcomes of various interventions, such as a specialist referral, CPAP therapy, and weight management, in individuals identified as high-risk. Finally, further investigation into the role of craniofacial structures in OSA, using a cephalometric analysis, may offer deeper insights into the anatomical contributions to the disorder.

## 5. Conclusions

This study demonstrates that both the STOP-Bang questionnaire and the Epworth sleepiness scale are effective tools for screening obstructive sleep apnea (OSA). STOP-Bang showed a strong predictive value by incorporating clinical and demographic risk factors such as BMI, age, and neck circumference, while Epworth effectively assessed the presence and impact of daytime sleepiness. Their simplicity, accessibility, and ease of administration make them suitable for use in dental settings, where they can support the early identification of individuals at risk for OSA and facilitate timely referral for further evaluation.

## Figures and Tables

**Table 1:** Demographic characteristics of the group.

	No	%
Age	61.25 ± 7.165 Years (Minimum Age: 42, Maximum Age: 75)	
Gender		
Female	91	41.0
Male	131	59.0
Residence		
Urban	151	68.0
Rural	71	32.0
Socio-economic level		
High socio-economic level	125	56.3
Medium socio-economic level	68	30.6
Low socio-economic level	29	13.1
Distribution of participants diagnosed with OSA		
No	190	85.6
Yes	32	14.4
Distribution of participants according to BMI		
Normal weight (<25 kg/m^2^)	1	.5
Overweight (25–30 kg/m^2^)	75	33.8
Obese (>30 kg/m^2^)	146	65.8
Distribution of participants according to associated pathologies		
No	15	6.8
Yes	207	93.2

**Table 2:** Distribution of participants’ responses to the STOP-Bang questionnaire according to gender, BMI, and associated pathologies.

		Gender		BMI			Associated Pathologies	
		Female	Male	Normal Weight	Increased Weight	Obese	No	Yes
You snore loudly	No	33.0%	22.9%	100.0%	46.7%	16.4%	100.0%	21.7%
	Yes	67.0%	77.1%		53.3%	83.6%		78.3%
*p*		0.097		0.000			0.000	
You are tired, exhausted, or sleepy during the day	No	31.9%	38.9%		74.7%	16.4%	80.0%	32.9%
	Yes	68.1%	61.1%	100.0%	25.3%	83.6%	20.0%	67.1%
*p*		0.281		0.000			0.000	
You have sleep apnea	No	49.5%	45.0%		74.7%	32.9%	80.0%	44.4%
	Yes	50.5%	55.0%	100.0%	25.3%	67.1%	20.0%	55.6%
*p*		0.517		0.000			0.008	
You have high blood pressure	No	60.4%	49.6%	100.0%	60.0%	50.7%	86.7%	51.7%
	Yes	39.6%	50.4%		40.0%	49.3%	13.3%	48.3%
*p*		0.112		0.275			0.009	
You are over 50 years old	No	2.2%	8.4%	100.0%	16.0%		86.7%	
	Yes	97.8%	91.6%		84.0%	100.0%	13.3%	100.0%
*p*		0.053		0.000			0.000	
Circumference <40 cm		52.7%	64.9%	100.0%	86.7%	45.9%	100.0%	57.0%
Circumference >41 cm		47.3%	35.1%		13.3%	54.1%		43.0%
*p*		0.070		0.000			0.001	
Low risk of obstructive sleep apnea: “Yes” to 0–2 questions		15.4%	16.8%	100.0%	46.7%		86.7%	11.1%
Medium risk of obstructive sleep apnea: “Yes” to 3–4 questions		27.5%	32.8%		41.3%	25.3%		32.9%
Increased risk of obstructive sleep apnea: “Yes” to 5–8 questions		57.1%	50.4%		12.0%	74.7%	13.3%	56.0%
*p*		0.598		0.000			0.001	

**Table 3:** Estimation of the chances of developing sleep apnea according to age, BMI, and classification into a risk group using the STOP-Bang questionnaire.

RISK		B	Std. Error	Wald	Sig.	Exp(B)	95% Confidence Interval for Exp(B)	
							Lower Bound	Upper Bound
Low risk of obstructive sleep apnea	Intercept	−1.360	0.208	42.690	0.000			
	[age2 = 1.00]	1.697	0.621	7.453	0.006	5.455	1.614	18.441
Medium risk of obstructive sleep apnea	Intercept	−0.633	0.160	15.705	0.000			
	[age2 = 1.00]	1.103	0.592	3.471	0.062	3.013	0.944	9.616
Medium risk of obstructive sleep apnea	Intercept	−1.080	0.190	32.245	0.000			
	[Body mass index = 1]	0.529	0.000			1.698	1.698	1.698
	[Body mass index = 2]	2.317	0.424	29.901	0.000	10.147	4.422	23.283

**Table 4:** Distribution of participants’ responses to the Epworth questionnaire according to gender, BMI, and associated pathologies.

	Gender		IMC			Associated Pathologies	
	Female	Male	Normal Weight	Increased Weight	Obese > 30 kg/m^2^	No	Yes
Sitting and reading a book or a newspaper							
Never doze/fall asleep	2.2%	8.4%	100.0%	16.0%		86.7%	
Low probability of dozing/falling asleep	13.2%	24.4%		58.7%			21.3%
Moderate likelihood of dozing/falling asleep	62.6%	38.2%		25.3%	60.3%		51.7%
High probability of dozing/falling asleep	22.0%	29.0%			39.7%	13.3%	27.1%
*p*	0.002		0.000			0.000	
Watching a show on TV							
Never doze/fall asleep	2.2%	8.4%	100.0%	16.0%		86.7%	
Low probability of dozing/falling asleep		16.0%		28.0%			10.1%
Moderate likelihood of dozing/falling asleep	48.4%	38.9%		56.0%	36.3%		45.9%
High probability of dozing/falling asleep	49.5%	36.6%			63.7%	13.3%	44.0%
*p*	0.000		0.000			0.000	
Sitting, inactive, in a public place (cinema, theatre, waiting room)							
Never doze/fall asleep	2.2%	24.4%	100.0%	44.0%		86.7%	10.1%
Low probability of dozing/falling asleep	2.2%	16.0%		25.3%	2.7%		11.1%
Moderate likelihood of dozing/falling asleep	78.0%	46.6%		30.7%	74.7%	13.3%	62.8%
High probability of dozing/falling asleep	17.6%	13.0%			22.6%		15.9%
*p*	0.000		0.000			0.000	
Passenger in a vehicle for a trip of at least 1 h							
Never doze/fall asleep	1.1%	8.4%	100.0%	14.7%		80.0%	
Low probability of dozing/falling asleep	3.3%	32.1%		54.7%	2.7%	6.7%	21.3%
Moderate likelihood of dozing/falling asleep	35.2%	24.4%			43.8%	13.3%	30.0%
High probability of dozing/falling asleep	60.4%	35.1%		30.7%	53.4%		48.8%
*p*	0.000			0.00		0.000	
Sitting in bed, after lunch							
Never doze/fall asleep	2.2%	8.4%	100.0%	16.0%		86.7%	
Low probability of dozing/falling asleep	13.2%	24.4%		58.7%			21.3%
Moderate likelihood of dozing/falling asleep	62.6%	38.2%		25.3%	60.3%		51.7%
High probability of dozing/falling asleep	22.0%	29.0%			39.7%	13.3%	27.1%
*p*	0.002		0.000			0.000	
Sitting and talking to someone							
Never doze/fall asleep	56.0%	58.8%		90.7%	41.1%	80.0%	56.0%
Low probability of dozing/falling asleep	41.8%	31.3%	100.0%	9.3%	48.6%	20.0%	36.7%
Moderate likelihood of dozing/falling asleep	2.2%	9.9%			10.3%		7.2%
High probability of dozing/falling asleep	56.0%	58.8%		90.7%	41.1%	80.0%	56.0%
*p*	0.039		0.000			0.167	
Seated, after a meal without alcoholic beverages							
Never doze/fall asleep	31.9%	32.1%	100.0%	58.7%	17.8%	93.3%	27.5%
Low probability of dozing/falling asleep	30.8%	32.8%		41.3%	27.4%	6.7%	33.8%
Moderate likelihood of dozing/falling asleep	37.4%	35.1%			54.8%		38.6%
High probability of dozing/falling asleep	31.9%	32.1%	100.0%	58.7%	17.8%	93.3%	27.5%
*p*	0.929		0.000			0.000	
Driving a car during a traffic stop lasting a few minutes							
Never doze/fall asleep	33.0%	41.2%	100.0%	72.0%	19.9%	100.0%	33.3%
Low probability of dozing/falling asleep	45.1%	35.9%		28.0%	45.9%		42.5%
Moderate likelihood of dozing/falling asleep	22.0%	22.9%			34.2%		24.2%
*p*	0.345		0.000		0.000		

## Data Availability

Data are contained within the article.
